# Efficacy of second-line chemotherapy after treatment with gemcitabine plus nab-paclitaxel or FOLFIRINOX in patients with metastatic pancreatic cancer

**DOI:** 10.1038/s41598-023-46924-0

**Published:** 2023-11-08

**Authors:** Masaru Fukahori, Yoshinobu Okabe, Mototsugu Shimokawa, Taiga Otsuka, Futa Koga, Yujiro Ueda, Junichi Nakazawa, Azusa Komori, Satoshi Otsu, Shiho Arima, Akitaka Makiyama, Hiroki Taguchi, Takuya Honda, Tomoyuki Ushijima, Keisuke Miwa, Taro Shibuki, Kenta Nio, Yasushi Ide, Norio Ureshino, Toshihiko Mizuta, Kenji Mitsugi, Tsuyoshi Shirakawa

**Affiliations:** 1 Division of Gastroenterology, Department of Medicine, Kurume University School of Medicine, 67 Asahi-Machi, Kurume-Shi, Fukuoka 830-0011 Japan; 2 Kyoto Innovation Center for Next Generation Clinical Trials and iPS Cell Therapy, Kyoto University Hospital, 54 Kawaharacho, Shogoin, Sakyo-Ku, Kyoto, 606-8507 Japan; 3 Clinical Research Institute, National Kyushu Cancer Center, 3-1-1 Notame, Minami-Ku, Fukuoka-Shi, Fukuoka 811-1395 Japan; 4 Department of Biostatistics, Yamaguchi University Graduate School of Medicine, 1-1-1 Minamikogushi, Ube-Shi, Yamaguchi 755-8505 Japan; 5 Department of Medical Oncology, Saga Medical Center Koseikan, 400 Kase-Machi, Saga-Shi, Saga 840-8571 Japan; 6 Department of Internal Medicine, Minato Medical Clinic, 3-11-3 Nagahama, Chuo-Ku, Fukuoka-Shi, Fukuoka 810-0072 Japan; 7 Department of Hepatobiliary and Pancreatology, Saga Medical Center Koseikan, 400 Kase-Machi, Saga-Shi, Saga 840-8571 Japan; 8 Department of Hematology and Oncology, Japanese Red Cross Kumamoto Hospital, 2-1-1 Nagamine-Minami, Higashi-Ku, Kumamoto-Shi, Kumamoto 861-8520 Japan; 9 Department of Medical Oncology, Kagoshima City Hospital, 37-1 Uearata-Cho, Kagoshima-Shi, Kagoshima 890-8760 Japan; 10 Department of Medical Oncology and Hematology, Faculty of Medicine, Oita University, 1-1 Idaigaoka, Hasama-Machi, Yufu-Shi, Oita 879-5593 Japan; 11 Digestive and Lifestyle Diseases, Kagoshima University Graduate School of Medical and Dental Sciences, 8-35-1 Sakuragaoka, Kagoshima-Shi, Kagoshima 890-8520 Japan; 12 Department of Hematology Oncology, Japan Community Healthcare Organization Kyushu Hospital, 1-8-1 Kishinoura, Yahatanishi-Ku, Kitakyushu-Shi, Fukuoka 806-8501 Japan; 13 Cancer Center, Gifu University Hospital, 1-1 Yanagido, Gifu-Shi, Gifu 501-1194 Japan; 14 Department of Gastroenterology, Saiseikai Sendai Hospital, 2-46 Harada-Cho, Satsumasendai-Shi, Kagoshima 895-0074 Japan; 15 Department of Gastroenterology, Imamura General Hospital, 11-23 Kamoike-Shinmachi, Kagoshima-Shi, Kagoshima 890-0064 Japan; 16 Department of Gastroenterology and Hepatology, Nagasaki University Graduate School of Biomedical Sciences, 1-7-1 Sakamoto, Nagasaki-Shi, Nagasaki 852-8501 Japan; 17 Multidisciplinary Treatment Cancer Center, Kurume University Hospital, 67 Asahi-Machi, Kurume-Shi, Fukuoka 830-0011 Japan; 18 Department of Internal Medicine, Imari Arita Kyoritsu Hospital, 860 Ninose-Ko, Arita-Cho, Nishi-Matsuura-Gun, Saga 849-4193 Japan; 19 Department of Hepatobiliary and Pancreatic Oncology, National Cancer Center Hospital East, 6-5-1 Kashiwanohara, Kashiwa-Shi, Chiba 277-8577 Japan; 20 Department of Medical Oncology, Sasebo Kyosai Hospital, 10-17 Shimanji-Cho, Sasebo-Shi, Nagasaki 857-8575 Japan; 21 Department of Medical Oncology, Hamanomachi Hospital, 3-3-1 Nagahama, Chuo-Ku, Fukuoka-Shi, Fukuoka 810-8539 Japan; 22 Department of Internal Medicine, Karatsu Red Cross Hospital, 2430 Watada, Karatsu-Shi, Saga 847-8588 Japan; 23 Department of Medical Oncology, Kimitsu Chuo Hospital, 1010 Sakurai, Kisarazu-Shi, Chiba 292-8535 Japan; 24 Department of Internal Medicine, Fujikawa Hospital, 1-2-6 Matsubara, Saga-Shi, Saga 840-0831 Japan; 25 Department of Medical Oncology, Fukuoka Wajiro Hospital, 2-2-75 Wajirogaoka, Higashi-Ku, Fukuoka-Shi, Fukuoka 811-0213 Japan; 26 Department of Internal Medicine, Karatsu Higashi-Matsuura Medical Association Center, 2566-11 Chiyoda-machi, Karatsu-Shi, Saga 847-0041 Japan

**Keywords:** Cancer, Gastroenterology, Medical research, Oncology

## Abstract

First-line chemotherapy for patients with metastatic pancreatic cancer (MPC) includes gemcitabine plus nab-paclitaxel (GnP) and FOLFIRINOX (FFX). However, the efficacy of second-line chemotherapy and the role of combination chemotherapy in clinical practice is still unknown. Data was gathered from 14 hospitals in the Kyushu area of Japan from December 2013 to March 2017. The median overall survival (mOS) from second-line treatment was contrasted between patients who received second-line chemotherapy (CT group) and those who received the best supportive care (BSC group). Furthermore, the mOS of combination chemotherapy was compared to mono chemotherapy in the CT group. To control possible bias in the selection of treatment, we performed a propensity score-adjusted analysis. A total of 255 patients received GnP or FFX as first-line chemotherapy. There were 156 in the CT group and 77 in the BSC group of these. The CT group had a significantly longer mOS than the BSC group (5.2 vs. 2.6 months; adjusted hazard ratio (HR) 0.38; 95% CI 0.27–0.54). In the CT group, 89 patients received combination chemotherapy while 67 received mono chemotherapy. The mOS did not differ significantly between the combination and mono chemotherapy groups (5.5 vs. 4.8 months; adjusted HR 0.88; 95% CI 0.58–1.33). Among patients with MPC receiving second-line treatment, the CT group had a significantly longer mOS than the BSC group, but combination chemotherapy conferred no improvement in survival compared to mono chemotherapy.

## Introduction

Pancreatic cancer has a poor prognosis and is having an increasing impact on cancer-related mortality in Japan and globally^1^. Pancreatic cancer is estimated to cause 40,000 new diagnoses and 33,000 deaths in Japan each year^2^. This disease is an outlier in the overall trend of decreasing cancer-related mortality. The five-year overall survival rate for locally advanced pancreatic cancer was 6.1%, while it was only 1.3% for metastatic disease^3^. For the past two decades, gemcitabine-based chemotherapies have been the standard of care for patients with advanced and metastatic pancreatic cancer^4,5^. Furthermore, two active combination regimens, FOLFIRINOX (a combination of oxaliplatin, folinic acid, irinotecan, and fluorouracil) (FFX) and gemcitabine plus nab-paclitaxel (GnP), have garnered acceptance as first-line treatment^6–8^

Although these active regimens are available following the failure of first-line therapy, there is little evidence of their efficacy in second-line treatment. Despite the unmet medical need for evidence-based second-line therapies, only a few pivotal phase III trials have been performed^9–11^. In clinical practice, the regimen used for first-line therapy, as well as the patient’s Eastern Cooperative Oncology Group performance status (ECOG-PS) and organ function, influence the choice of second-line treatment. Active combination regimens are recommended by the ASCO clinical practice guidelines for patients with an ECOG-PS of 0 or 1 and a favorable comorbidity profile^12^. However, until the recent approval of nanoliposomal irinotecan (nal-IRI), significant data to support the use of combination regimens in a second-line setting were insufficient.

We evaluated the efficiency of second-line treatment after FFX or GnP first-line chemotherapy for metastatic pancreatic cancer (MPC). First, the efficacy of second-line chemotherapy was contrasted with best supportive care (BSC). Second, patient survival was contrasted following second-line mono chemotherapy or combination chemotherapy.

## Results

### Patients’ characteristics

**Figure 1:**
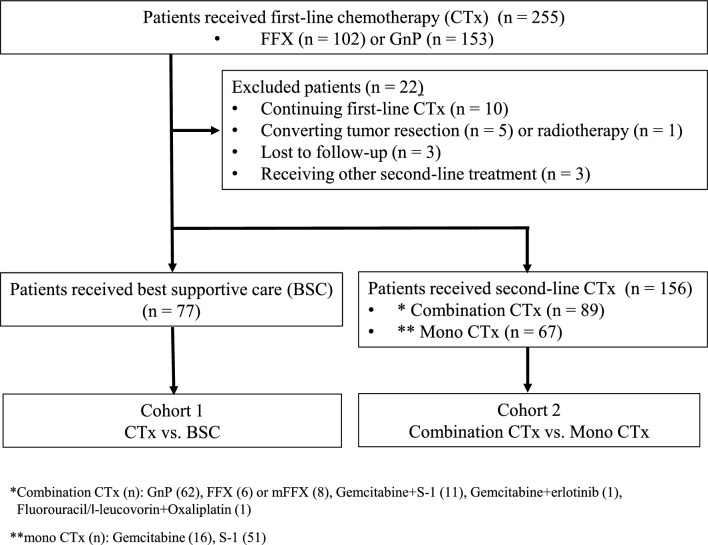
A flow diagram of this study. Combination. CTx: Gemcitabine plus Nab-Paclitaxel, FOLFIRINOX, Gemcitabine plus S-1, Gemcitabine plus Erlotinib, Fluorouracil/l-leucovorin plus oxaliplatin, Mono CTx: Gemcitabine, S-1.

From December 2013 to March 2017, 102 and 153 patients with MPC initially received FFX and GnP, respectively. 156 of these patients received second-line chemotherapy, while 77 received BSC. In the second-line group, 89 patients received combination chemotherapy [GnP (62), gemcitabine plus S-1 (11), FFX (6) and modified FFX (8), gemcitabine plus Erlotinib (1) and fluorouracil/ l-leucovorin plus oxaliplatin (1)] and 67 patients received monochemotherapy [gemcitabine (16), and S-1 (51)] (Fig. 1). Patients’ baseline characteristics are in depicted Table 1 for analysis 1 and Table 2 for analysis 2.

**Table 1:** Patient demographics and characteristics in Cohort 1.

		Original data				Data after propensity score matching			
		Second-line chemotherapy n = 156	BSC n = 77	*P*	SMD	Second-line chemotherapy n = 98	BSC n = 61	*P*	SMD
Age (years)	Years, median (range)	64 (35–86)	66 (39–86)	0.03	0.28	64 (35–86)	66 (39–78)	0.34	0.15
Sex, n (%)	Male	91 (58)	51 (66)	0.25	0.16	55 (56)	41 (67)	0.16	0.23
	Female	65 (42)	26 (34)			43 (44)	20 (33)		
ECOG PS, n (%)	0	110 (71)	35 (45)	< 0.01	0.56	60 (61)	35 (57)	0.75	0.19
	1	39 (25)	33 (43)			31 (32)	23 (38)		
	≥ 2	7 (4)	9 (12)			7 (7)	3 (5)		
Previous surgical resection, n (%)		19 (12)	18 (23)	0.03	0.30	16 (16)	13 (21)	0.43	0.13
Tumor location, n (%)	Head	74 (47)	37 (48)	0.82	0.09	46 (47)	29 (48)	0.84	0.1
	Body	48 (31)	21 (27)			31 (32)	17 (28)		
	Tail	34 (22)	19 (25)			21 (21)	15 (25)		
Histology, n (%)	Adenocarcinoma	136 (87)	60 (78)	0.10	0.28	86 (88)	46 (75)	0.11	0.34
	Others	2 (1)	4 (5)			2 (2)	4 (7)		
	Unknown	18 (12)	13 (17)			10 (10)	11 (18)		
Number of metastatic sites, n (%)	1	97 (62)	44 (57)	0.46	0.10	60 (61)	36 (59)	0.78	0.05
	≥ 2	59 (38)	33 (43)			38 (39)	25 (41)		
Ascites, n (%)		29 (19)	23 (30)	0.052	0.27	19 (19)	13 (21)	0.77	0.05
Albumin level (g/dL)	Median (range)	3.9 (2.5–4.8)	3.6 (2.2–4.8)	< 0.01	0.50	3.9 (2.5–4.8)	3.7 (2.2–4.8)	0.11	0.28
LDH level (U/L)	Median (range)	176 (99–923)	194 (74–1320)	0.02	0.24	178 (122–723)	195 (74–1320)	0.12	0.24
CRP level (mg/dL)	Median (range)	0.28 (0.01–8.88)	0.90 (0.01–17.00)	0.02	0.39	0.42 (0.01–8.88)	0.86 (0.01–15.80)	0.27	0.30
CEA level (ng/mL)	Median (range)	5.4 (0.4–626.6)	7.0 (0.8–369.8)	0.16	0.07	5.4 (0.4–626.6)	7.4 (0.8–164.3)	0.25	0.19
CA19-9 level (U/mL)	Median (range)	1122 (1–6,554,100)	835 (1–4,690,400)	0.94	0.02	1287 (1–6,554,100)	752 (1–277,056)	0.87	0.16
Neutrophil-to-lymphocyto ratio	Median (range)	2.05 (0.09–15.17)	2.61 (0.24–21.96)	0.09	0.25	2.31 (0.09–15.17)	2.61 (0.36–21.96)	0.69	0.05
First-line chemotherapy, n (%)	FFX	79 (51)	15 (19)	< 0.01	0.69	34 (35)	15 (25)	0.18	0.22
	GnP	77 (49)	62 (81)			64 (65)	46 (75)		
Withdrawal reason, n (%)	Progression	137 (88)	55 (71)	< 0.01	0.42	83 (85)	46 (75)	0.35	0.23
	Adverse events	16 (10)	18 (23)			12 (12)	12 (20)		
	Others	3 (2)	4 (5)			3 (3)	3 (5)		
Duration of first-line chemotherapy	Months, median (range)	5.6 (0.4–30.8)	3.5 (0.2–22.2)	0.02	0.23	4.8 (0.4–19.8)	3.6 (0.2–22.2)	0.28	0.06

**Table 2:** Patient demographics and characteristics in Cohort 2.

		Original data				Data after propensity score matching			
		Combination therapy n = 89	Monotherapy n = 67	*P*	SMD	Combination therapy n = 35	Monotherapy n = 35	*P*	SMD
Age (years)	Years, median (range)	62 (35–79)	68 (48–86)	< 0.01	0.57	63 (40–79)	64 (49–79)	0.23	0.36
Sex, n (%)	Male	49 (55)	42 (63)	0.34	0.16	20 (57)	23 (66)	0.46	0.18
	Female	40 (45)	25 (37)			15 (43)	12 (34)		
ECOG PS, n (%)	0	67 (75)	43 (64)	0.02	0.49	26 (74)	27 (77)	0.53	0.27
	1	22 (25)	17 (25)			9 (26)	7 (20)		
	≥ 2	0	7 (11)			0	1 (3)		
Previous surgical resection, n (%)		11 (12)	8 (12)	0.94	0.01	5 (14)	5 (14)	1.00	< 0.01
Tumor location, n (%)	Head	40 (45)	34 (51)	0.45	0.21	14 (40)	19 (54)	0.33	0.36
	Body	31 (35)	17 (25)			10 (29)	10 (29)		
	Tail	18 (20)	16 (24)			11 (31)	6 (17)		
Histology, n (%)	Adenocarcinoma	79 (89)	57 (85)	0.79	0.11	31 (89)	30 (86)	0.92	0.10
	Others	1 (1)	1 (1)			1 (3)	1 (3)		
	Unknown	9 (10)	9 (13)			3 (9)	4 (11)		
Number of metastatic sites, n (%)	1	57 (64)	40 (60)	0.58	0.09	19 (54)	23 (66)	0.33	0.24
	≥ 2	32 (36)	27 (40)			16 (46)	12 (34)		
Ascites, n (%)		17 (19)	12 (18)	0.85	0.03	5 (14)	4 (11)	0.72	0.09
Albumin level (g/dL)	Median (range)	3.9 (2.8–4.8)	3.9 (2.5–4.7)	0.31	0.22	3.9 (2.8–4.5)	3.9 (2.5–4.4)	0.72	0.11
LDH level (U/L)	Median (range)	181 (99–923)	169 (122–328)	0.04	0.30	177 (147–923)	167 (122–328)	0.09	0.44
CRP level (mg/dL)	Median (range)	0.24 (0.01–8.88)	0.38 (0.01–7.26)	0.85	0.14	0.28 (0.01–8.88)	0.14 (0.02–6.60)	0.28	0.15
CEA level (ng/mL)	Median (range)	6.0 (0.4–364.2)	4.1 (0.7–626.6)	0.88	0.25	6.0 (0.4–304.5)	3.4 (0.7–336.7)	0.50	0.18
CA19-9 level (U/mL)	Median (range)	987 (1–6,554,100)	1232 (1–590,340)	0.84	0.15	891 (1–1,242,609)	717 (2–96,693)	0.25	0.26
Neutrophil-to-lymphocyto ratio	Median (range)	1.96 (0.09–7.57)	2.31 (0.59–15.17)	0.10	0.39	2.64 (0.65–7.57)	1.91 (0.59–15.00)	0.54	0.09
First-line chemotherapy, n (%)	FFX	66 (74)	13 (29)	< 0.01	1.31	12 (34)	12 (34)	1.00	< 0.01
	GnP	23 (26)	54 (81)			23 (66)	23 (66)		
Withdrawal reason, n (%)	Progression	83 (93)	54 (81)	0.057	0.38	35 (100)	26 (74)	< 0.01	0.83
	Adverse events	5 (6)	11 (16)			0	7 (20)		
	Others	1 (1)	2 (3)			0	2 (6)		
Duration of first-line chemotherapy	Months, median (range)	5.4 (0.4–30.8)	5.8 (0.4–18.8)	0.61	0.03	5.8 (0.9–30.8)	5.8 (0.4–10.6)	0.31	0.45

### Analysis 1

Table 1 compares patient survival after first-line chemotherapy and then BSC (Cohort 1). The BSC group had more patients with poor performance status and lower albumin concentrations in cohort 1.

**Figure 2:**
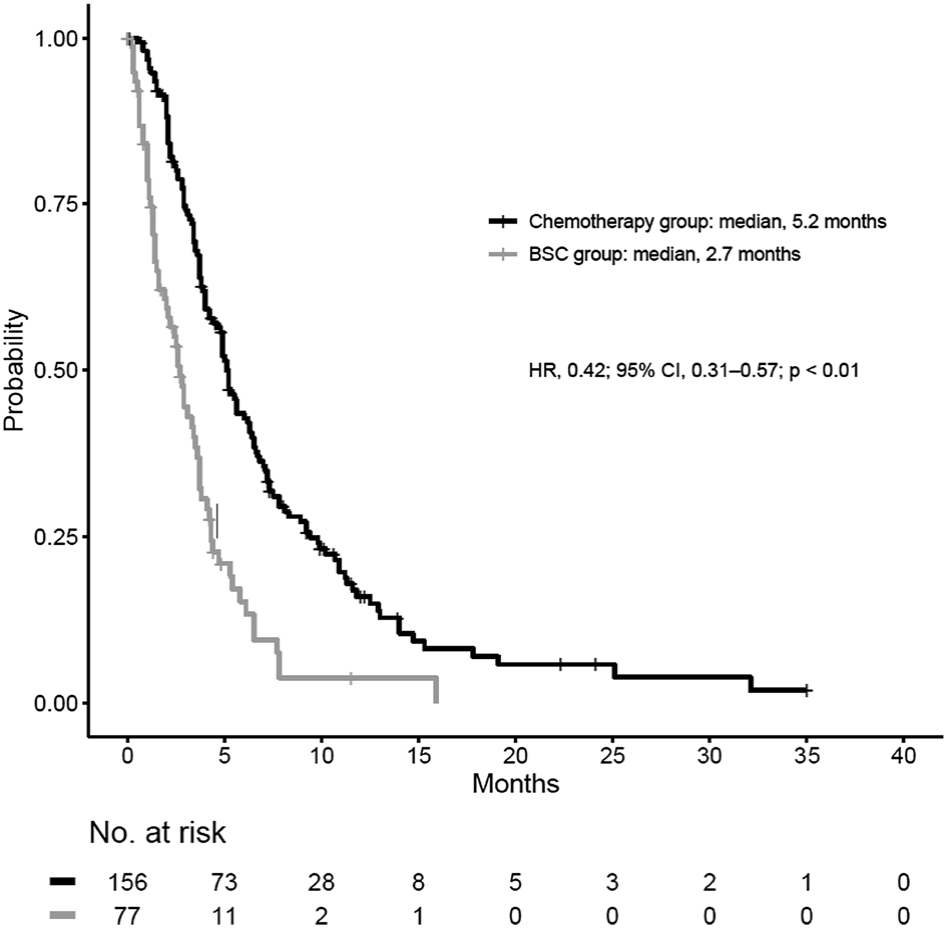
Kaplan–Meier curves comparing patients’ survival after second-line treatment with chemotherapy or BSC (Cohort1).

According to a provisional analysis of the fundamental dataset, the median overall survival (mOS) (second-line) for the chemotherapy group was 5.2 months and 2.7 months for the BSC group (HR 0.42; 95% CI 0.31–0.57; *P* < 0.01) (Fig. 2). The mOS after first-line treatment for the chemotherapy group was 11.7 months and 6.9 months for the BSC group (HR 0.50; 95% CI 0.37–0.68; *P* < 0.01).

In the propensity score-adjusted analysis, mOS (after second-line treatment) was 5.2 months for the chemotherapy group and 2.6 months for the BSC group (HR 0.38; 95% CI 0.27–0.54; *P* < 0.01) The mOS from the first-line was 11.5 months for the chemotherapy group and 7.8 months for the BSC group (HR 0.56; 95% CI 0.41–0.78; *P* < 0.01). The logistic regression model’s c-statistics value for calculating the propensity score was 0.75 (95% CI 0.68–0.82).

Following propensity score-matching, 98 chemotherapy group patients were matched with 61 BSC group patients (Table 1). The mOS after second-line treatment for the chemotherapy group was 5.5 months and 2.5 months for the BSC group (HR 0.42; 95% CI 0.29–0.61; *P* < 0.01). In the IPTW analysis, the mOS after second-line treatment for the chemotherapy group was 5.5 months and 2.5 months for the BSC group (HR 0.40; 95% CI 0.28–0.58; *P* < 0.01).

### Analysis 2

**Figure 3:**
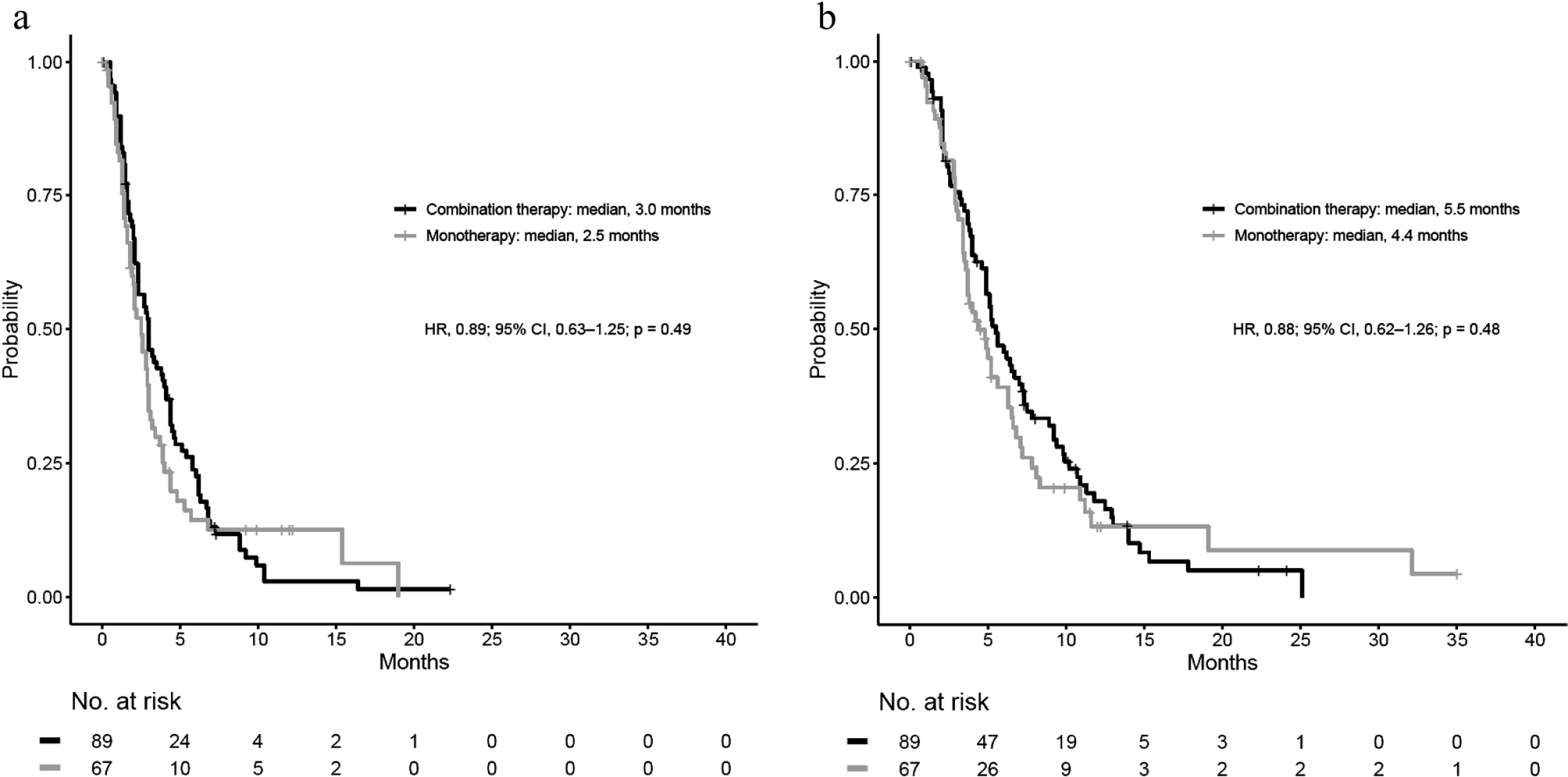
Kaplan–Meier curves comparing (**a**) PFS and (**b**) OS in patients who received second-line combination chemotherapy or mono chemotherapy, according to a preliminary analysis of the fundamental dataset (Cohort 2).

Table 2 contrasts patient survival between those who received combination chemotherapy and those who received mono chemotherapy (Cohort 2). In cohort 2, the mono chemotherapy group included more elderly patients. The median PFS (mPFS) (after second-line treatment) for the mono chemotherapy group was 2.5 months and 3.0 months for the combination chemotherapy group (HR 0.89; 95% CI 0.63–1.25; *P* = 0.49) (Fig. 3a). A preliminary analysis of the fundamental dataset revealed that the mOS (after second-line treatment) for the combination chemotherapy group was 5.5 months and 4.4 months for the mono chemotherapy group (HR 0.88; 95% CI 0.62–1.26; *P* = 0.48) (Fig. 3b). After first-line treatment, the mOS for the combination chemotherapy group was 12.1 months and 11.4 months for the mono chemotherapy group (HR 0.98; 95% CI 0.69–1.39; *P* = 0.90).

In the propensity score-adjusted analysis, the mPFS (after second-line treatment) was 2.6 months for the mono chemotherapy group and 3.0 months for the combination chemotherapy group (HR 0.98; 95% CI 0.66–1.46; *P* = 0.92). The mOS (after second-line treatment) was 5.5 months for the combination chemotherapy group and 4.8 months for the mono chemotherapy group (HR 0.88; 95% CI 0.58–1.33; *P* = 0.53). After first-line treatment, the mOS after first-line treatment was 12.3 months for the combination chemotherapy group and 11.1 months for the mono chemotherapy group (HR 0.86; 95% CI 0.56–1.31; *P* = 0.47). The logistic regression model’s c-statistics value for calculating the propensity score was 0.82 (95% CI 0.76–0.89).

After propensity score-matching, 35 patients in the combination chemotherapy group were matched with 35 patients in the mono chemotherapy group (Table 2). The mPFS (after second-line treatment) was 2.95 months for the mono chemotherapy group and 3.0 months for the combination chemotherapy group (HR 1.19; 95% CI 0.71–1.99; *P* = 0.51). The mOS from the second-line was 6.4 months for the combination chemotherapy group and 6.5 months for the mono chemotherapy group (HR 0.98; 0.56–1.71; *P* = 0.95). In IPTW analysis, the mOS from the second-line was 5.5 months for the combination chemotherapy group and 3.8 months for the mono chemotherapy group (HR 0.84; 95% CI 0.52–1.34; *P* = 0.45).

## Discussion

The survival period of patients with MPC remains very short because this disease is resistant to chemotherapy and progresses rapidly. The sequencing of therapeutic options in the second-line setting for patients with MPC remains a significant unfulfilled need. We examined the outcomes of second-line treatment using the cohort of patients who received FFX or GnP as a first-line therapy. First, we conducted a study that focused on comparing survival with chemotherapy versus BSC. Second, we compared survival following second-line combination chemotherapy or mono chemotherapy.

Most prior randomized control trials (RCT) did not compare active treatment with BSC. The first and only phase III RCT compared BSC to oxaliplatin, fluoropyrimidine (5-FU), and folic acid (FA) (OFF regimen) in 46 patients^9^. Although this study ended prematurely due to low enrollment, the OFF regimen was associated with significantly higher survival after second-line treatment compared with BSC (4.8 versus 2.3 months, *p* = 0.008).

In our study, 77 patients received BSC after FFX and GnP failed. There is no data comparing chemotherapy to BSC in the second-line treatment setting after refractory response to intensive chemotherapy regimens such as FFX and GnP. Survival of the second-line chemotherapy group was considerably longer than the BSC’s (mOS 5.2 versus 2.7 months, HR 0.42 (95% CI 0.31–0.57), *P* < 0.01). However, because these two groups may differ in terms of background factors after first-line treatment failure, a propensity score-adjustment analysis was performed before second-line treatment by selecting clinically meaningful variables. The adjusted results also revealed a statistically significant increase in mOS for patients receiving second-line chemotherapy versus those that chose the second-line BSC group (mOS 5.2 versus 2.6 months, HR 0.38 (95% CI 0.27–0.54), *P* < 0.01). These findings suggest that administering any second-line chemotherapy to patients who are considered chemotherapy tolerant will help to prolong survival.

As a criterion for selecting a regimen for second-line treatment, we confirmed the correlation between the adverse events that occurred during first-line treatment (Table 3). Analysis 1 showed that the BSC group had a higher incidence of grade 3 or higher anemia than did the second-line chemotherapy group (*P* = 0.04). In contrast, no differences in the frequency of neutropenia, febrile neutropenia, or gastrointestinal toxicity were observed between the groups, all of which had no impact on the transition to second-line treatment or BSC. Reports have shown that anemia is associated with chemotherapy resistance, with our study showing a similar trend^13^. For cases with grade 3 or higher anemia, depending on the situation after first-line treatment failure, BSC may be considered as a necessary option. In Analysis 2, no differences in adverse events occurring during first-line treatment were observed between the combination and monotherapy groups. For patients who exhibited severe peripheral neuropathy during first-line treatment, avoiding regimens that may cause peripheral neuropathy during second-line treatment may not decrease their QOL.

**Table 3:** Grade 3 or higher adverse events at first-line chemotherapy affecting ≥ 5% of patients.

		Cohort 1			Cohort 2		
		Second-line chemotherapy n = 156	BSC n = 77	*P*	Combination therapy n = 89	Monotherapy n = 67	*P*
Hematological, n (%)	Leukopenia	50 (32)	23 (30)	0.74	25 (28)	25 (37)	0.22
	Neutropenia	96 (62)	38 (49)	0.08	54 (61)	42 (63)	0.80
	Anemia	17 (11)	16 (21)	0.04	9 (10)	8 (12)	0.72
	Thrombocytopenia	16 (10)	10 (13)	0.53	6 (7)	10 (15)	0.10
Nonhematological, n (%)	Febrile neutropenia	17 (11)	10 (13)	0.64	9 (10)	8 (12)	0.72
	Anorexia	12 (8)	11 (14)	0.11	7 (8)	5 (8)	0.93
	Nausea	7 (5)	5 (7)	0.52	6 (7)	1 (2)	0.12
	Diarrhea	9 (6)	1 (1)	0.11	6 (7)	3 (5)	0.55
	Sensory neuropathy	15 (10)	7 (9)	0.90	5 (6)	10 (15)	0.051
	ALT increased	9 (6)	6 (8)	0.55	7 (8)	2 (3)	0.20
	Biliary tract infection	7 (5)	5 (7)	0.52	4 (5)	3 (5)	0.996

Correlation of adverse events during first-line treatment with the treatment details of cohort 1 and cohort 2.

Patient background data for Analysis 1 showed that the BSC group had a higher proportion of patients with a history of resection than did the chemotherapy group. We believe that there are two reasons for such a finding. The first reason for this is that in Japan, S-1, a fluoropyrimidine-based agent, is the standard of care for adjuvant chemotherapy, whereas gemcitabine-based regimens are selected for recurrence. When this regimen becomes ineffective, treatment options are limited and BSC may be considered. The second reason is that this study used a database from a period wherein the safety of FFX in second-line therapy was not well established. Therefore, FFX may have been less commonly selected among patients refractory to gemcitabine-based regimen.

The validity of second-line therapy for recurrent cases after surgery remains controversial. However, given that it is being carried out in clinical practice, we conducted an adjusted analysis. Regarding the validity of chemotherapy for postoperative cases, we are currently conducting a prospective investigation on the matter (NAPOLEON2 study).

In recurrent or advanced pancreatic cancer, the prognostic difference based on the background cannot be ignored. In a sub-analysis of the main study, survival data for metastatic PC (mPC) and recurrent (rPC) treated with FFX or GnP therapy as first-line treatment were analyzed, with our findings showing that rPC had a favorable prognosis. However, propensity score-matched analysis revealed no difference in prognosis between the two groups with chemotherapy^14^. However, the possibility that mPC and rPC contribute to prognosis cannot be ruled out in second-line treatment; hence, additional prognostic analyses were conducted. Notably, Analysis 1 showed no significant difference between the two groups, with mPC (n = 196) and rPC (n = 37) at 4.0 and 4.9 months, respectively (HR 0.78; 95% CI 0.52–1.16; *P* = 0.21). Similarly, Analysis 2 showed no significant difference between the two groups, with mPC (n = 137) and rPC (n = 19) at 5.1 and 8.9 months, respectively (HR 0.65; 95% CI 0.36–1.15; *P* = 0.14). In both Analysis 1 and Analysis 2, no significant differences were observed between the two groups.

Although nal-IRI and 5-FU/FA combination therapy is recommended for second-line treatment after a gemcitabine-based regimen fails, data regarding the efficacy of other combination regimens are lacking. A few pivotal phase III trials have been conducted. In a phase III RCT, the OFF regimen significantly improved survival in patients with gemcitabine refractory pancreatic cancer compared with a 5-FU/FA regimen; the benefit of additional oxaliplatin was also demonstrated (mOS 5.9 versus 3.3 months, *p* = 0.01, median time to progression 2.9 versus 2.0 months, *p* = 0.019)^10^. On the other hand, FOLFOX has been observed to be subordinate to 5-FU/FA for OS (median 6.1 versus 9.9 months, *p* = 0.02), but no difference was observed in PFS (median, 3.1 versus 2.9 months, *p* = 0.02)^15^; thus, leaving the significance of oxaliplatin in second-line therapy unclear. The NAPOLI-1 study, a pivotal phase III RCT, found that nal-IRI plus 5-FU/FA significantly improved survival compared with 5-FU/FA (median: 6.1 versus 4.2 months, *p* = 0.0012) and PFS (median, 3.1 versus 1.5 months, *p* = 0.00001) in patients previously treated with a gemcitabine-based regimen^11^. The mOS and mPFS in various phase III RCTs of second-line pancreatic cancer treatment is inconsistent, ranging from about 3.3 to 9.9 months and 1.5 to 3.1 months^9–11,15^. Most studies to date have evaluated second-line regimens following gemcitabine-based therapy failure. Furthermore, there is little evidence supporting the choice between combination chemotherapy or mono chemotherapy for patients who are refractory to FFX or GnP treatment.

We compared the survival of the second-line combination chemotherapy group with the second-line mono chemotherapy group. Remarkably, the combination chemotherapy group did not exert substantial variations in OS (median 5.5 versus 4.4 months, *p* = 0.48) or PFS (median 3.0 versus 2.5 months, *p* = 0.49) as compared to the mono chemotherapy group. Propensity score-adjustment analysis did exert significant differences in OS (median 5.5 versus 4.8 months, *p* = 0.53) or PFS (median 2.6 versus 3.0 months, *p* = 0.92). After the end of the second-line treatment, there were 85 evaluable patients in the combination chemotherapy group and 60 patients in the mono-chemotherapy group, respectively. Of these patients, 30 (35%) were in the combination chemotherapy group and 14 (23%) were in the mono-chemotherapy group. There was no significant difference between the two groups in terms of third-line chemotherapy transition (*p* = 0.12); however the clinical impact was undeniable (Supplemental Table 1).

Significant differences in patients’ backgrounds between the two groups were reported in age, ECOG-PS, and LDH. Patients in the mono chemotherapy group were inclined to be older and had lower ECOG-PS. Although second-line chemotherapy for MPC is expected to prolong survival, increasing the intensity may not necessarily result in increased longevity. Patients with MPC who have exerted poor response to first-line chemotherapy frequently present with poor ECOG-PS and a high symptom burden; thus, a combination chemotherapeutic approach may not be appropriate for patients in the salvage setting. Patients with a poor response, worsened ECOG-PS, and a high tumor burden is unlikely to benefit from intensive chemotherapy, and they may suffer more harm than benefit^16^.

In the current study, the survival period in patients receiving combination chemotherapy was comparable to that of patients receiving mono chemotherapy; however, there were some limitations. First, while we performed a propensity score-adjustment analysis, this is a retrospective study, which requires consideration of selection bias in treatment decisions. There were differences in age and ECOG-PS in patient background between the two groups of patients who received FFX and GnP in first-line chemotherapy^17^. In analysis 1, the BSC transfer rate was 16% in the FFX group as first-line chemotherapy and 45% in the GnP group as first-line chemotherapy. Furthermore, in analysis 2, the mono-chemotherapy transfer rate was 16% in the FFX group as first-line chemotherapy and 70% in the GnP group as first-line chemotherapy. Therefore, it is necessary to consider that patient background influenced the choice of the first-line chemotherapy regimen, which also affected the second-line treatment. From ACCORD11 and MPACT data, BSC transition rates were 53% and 62%, respectively^6,7^. Based on these results, the transition rate to second-line chemotherapy in this study was favorable. However, it is necessary to consider that patient background influenced the choice of first-line chemotherapy regimen, which also affected second-line treatment. This study did not reverse the results before and after the propensity score-adjusted analysis. In this regard, we must consider the possibility that the number of cases decreased after the propensity score-adjusted analysis, which could have affected the reliability of our results. If possible, the number of cases should have been added by extending the study period. However, the time when nal-IRI would become available was imminent in Japan, and it had been decided that a validated multidrug regimen would become the standard of care. In this study, the analysis was performed on the data before using nal-IRIs to eliminate bias.

Second, we have not investigated the role of the induction dose of the second-line regimens, the response rate, or adverse events that may have influenced the choice of mono chemotherapy or combination chemotherapy as the second-line regimen. Third, this study did not include a nal-IRI-based regimen as a second-line treatment, because it was not endorsed for use in Japan during the survey period. The NAPOLEON-2 is a prospective trial currently ongoing, which seeks to investigate the efficacy of combination chemotherapy (nal-IRI plus 5-FU/FA) as compared to mono chemotherapy (S-1); the results are currently awaited (UMIN000043939).

Conclusively, after comparing chemotherapy versus BSC, if the patient’s tolerability is tolerable, second-line chemotherapy for patients with MPC can be expected to prolong survival. Regarding the treatment options, combination chemotherapy does not always contribute to longer survival and that mono chemotherapy should also be considered a viable option.

## Methods

### Patients

This study was conducted as a multicenter retrospective study of patients with unresectable or recurrent pancreatic cancer who received GnP and FFX first-line chemotherapy (NAPOLEON study) by medical oncology and gastroenterology specialists practicing in Japan’s Kyushu region. The institutional review board of each participating institution authorized the NAPOLEON study. We reviewed the medical records of consecutive patients with unresectable or recurrent pancreatic cancer who began FFX or GnP as first-line chemotherapy from December 2013 to March 2017. We excluded patients with locally advanced diseases in this study.

### Treatment methods

As the regimens of the second-line chemotherapy aside GnP and gemcitabine plus S-1, FFX included original and modified FOLFIRINOX, gemcitabine plus Erlotinib, fluorouracil/ l-leucovorin plus oxaliplatin, gemcitabine, and S-1 were included in the analysis. Patients who received other second-line treatments (hepatic artery infusion, everolimus, or unknown) were excluded. Patients receiving two or more drug regimens were categorized as the combination chemotherapy group, while patients treated with a single drug regimen, gemcitabine or S-1, were classified as the mono chemotherapy group. The induction dose for the second-line chemotherapy was left to the physicians’ discretion.

### Assessment

The primary goal of the study was overall survival (OS). Secondary endpoints included progression-free survival (PFS). OS was calculated from the administration date of second-line chemotherapy to the date of death from any cause or censored at the final follow-up examination. BSC was thought to begin after the first sign of disease progression following treatment first-line chemotherapy. PFS was calculated from the date of second-line chemotherapy administration to the date of progression or death from any cause, whichever occurred first or was censored at the final follow-up examination.

### Statistical analysis

We initially performed preliminary analyses of the original cohort (fundamental dataset) to estimate the effects of treatment. For continuous data, the Mann–Whitney test was used, and for categorical data, the *Χ*^2^-test was used. OS and PFS were estimated using the Kaplan–Meier method and comparisons of the probability of survival were conducted using the log-rank test and the Cox proportional hazards model. The hazard ratio (HR) was represented with a 95% confidence interval (95% CI). Differences with *P* < 0.05 values were considered substantial. We used propensity score-adjusted analyses to control for potential selection bias linked with nonrandomization. The propensity score was calculated using the logistic regression model using the following covariates: age, ECOG-PS, duration of first-line chemotherapy, resection of the primary lesion, first-line regimen, reason for first-line chemotherapy withdrawal for second-line chemotherapy, performance versus BSC (analysis 1) and first-line regimen, age, ECOG-PS for combination chemotherapy versus mono chemotherapy (analysis 2). The covariates were selected based on the pretreatment background characteristics of the primary dataset, which were utilized by clinicians when deciding on treatment. We also refer to the suggested baseline variables, according to the international consensus statement used to examine the efficacy and safety of systemic therapy for unresectable pancreatic cancer^18^. A propensity score-matched analysis and inverse probability of treatment weighting (IPTW) were used to establish sensitivity to evaluate the associations between treatment selection and endpoints. The logistic regression model’s predictive accuracy was evaluated using c-statistics. The Cox proportional hazard model was applied to the fundamental dataset with propensity scores and treatment groups as covariates, and a propensity score-adjusted analysis was performed. Propensity score-matched analysis utilized a two-to-one for analysis 1 and a one-to-one for analysis 2 nearest-neighbor matching with the caliper set to 0.2. Statistical analyses were conducted using R ver. 3.5.3 (R Foundation for Statistical Computing, Vienna, Austria).

### Ethics approval and consent to participate

This study was conducted in accordance with the ethical guideline of the Declaration of Helsinki and was centrally approved by the Institutional review board of Saga Medical Center Koseikan (study ID 17-09-01-02), and also approved by the Institutional Review Boards or Ethics Committee of following institutions: Imari Arita Kyoritsu Hospital, Japanese Red Cross Kumamoto Hospital, Kagoshima City Hospital, Oita University Hospital, Kagoshima University Hospital, Kurume University Hospital, Japan Community Healthcare Organization Kyushu Hospital, Saiseikai Sendai Hospital, Nagasaki University Hospital, Hamanomachi Hospital, Sasebo Kyosai Hospital, Karatsu Red Cross Hospital and Fukuoka Wajiro Hospital prior to the study. Because this study was a retrospective observational study carried out in Japan, informed consent was obtained using the opt-in/opt-out approach according to each participating institution’s policy.

### Supplementary Information

Supplementary Table 1.

## Data Availability

All data generated or analyzed in this article are stored in a secured research database. They are not publicly available; however, are available through the corresponding author upon reasonable request.

## Code availability

We used R ver. 4.2.0 (R Foundation for Statistical Computing, Vienna, Austria) for statistical analyses.
