# Supplementary material for: Efficacy of second-line chemotherapy after treatment with gemcitabine plus nab-paclitaxel or FOLFIRINOX in patients with metastatic pancreatic cancer
Source: Sci Rep. 2023 Nov 8;13:19399. doi: 10.1038/s41598-023-46924-0 (PMC10632451; doi:10.1038/s41598-023-46924-0)
Supplement: Supplementary file 1 [file 41598_2023_46924_MOESM1_ESM.docx]

Supplement table 1. Treatment status after the second-line chemotherapy

At the end of second-line chemotherapy

		Combination CTx (n= 89)	Mono CTx (n= 67)
Stopped treatment	PD	73	49
	AE	9	10
	Patient request	3	0
	other	0	1
Censoring		4	7
evaluable patient		85	60

Third-line treatment status

BSC (%)	55 (65)	46 (77)	P
Third-line chemotherapy (%)	30 (35)	14 (23)	0.1

Third-line regimen

S-1	20	3
Gemcitabine+ S-1	4	2
modified FOLFIRINOX	2	3
Gemcitabine	2	2
Gemcitabine+ elrotinib	1	0
Gemcitabine+ nab-Paclitaxel	0	4
Unknown	1	0
